# A newly discovered *Hystrix primigenia* specimen from the Kemiklitepe collection at Ege University Natural History Museum: insights into paleobiogeography in Eurasia

**DOI:** 10.1111/1749-4877.12820

**Published:** 2024-04-03

**Authors:** Kazım HALAÇLAR, Paul RUMMY, Serdar MAYDA, Tao DENG

**Affiliations:** ^1^ Natural History Application and Research Centre Ege University Izmir Türkiye; ^2^ Key Laboratory of Vertebrate Evolution and Human Origins, Institute of Vertebrate Paleontology and Paleoanthropology Chinese Academy of Sciences Beijing People's Republic of China; ^3^ Department of Biology, Faculty of Sciences Ege University Izmir Türkiye

**Keywords:** *Hystrix*, Kemiklitepe, late Miocene, paleobiogeography, Türkiye

## Abstract

Porcupines, members of the Hystricidae family, represent a unique group of herbivorous mammals. This study details the identification of a newly discovered mandible fragment of *Hystrix primigenia*, along with a right cheek tooth series from the middle Turolian Kemiklitepe‐A fossil locality. While *Hystrix* fossils are found in numerous localities, the materials are often limited to a few dental fragments or isolated teeth, posing challenges to systematic investigations. The examination of this lower tooth series prompted a comprehensive review of all *H. primigenia* findings across Eurasia, shedding light on its adaptive characteristics over time and space. Our paleobiogeographical analysis indicates the absence of *H. depereti* in Late Miocene Türkiye, while the dispersal range of *H. primigenia* is broader than that of *H. depereti* in Eurasia. Additionally, the study delves into the discussion of *H. primigenia* and *H. depereti* findings in Eurasia, ultimately refining the categorization of Late Miocene *Hystrix* discoveries in Türkiye to two species: *H. primigenia* and *H. kayae*. Our review suggests the possibility of an additional *H. kayae* finding from Samos, Greece.

## Graphical abstract

Our paleobiogeographical analysis reveals the absence of *Hystrix depereti* in Late Miocene Türkiye, parallel with the broader dispersal range of *Hystrix primigenia* in Eurasia. Examining *H. primigenia* and *H. depereti* findings in Eurasia, our study refines Late Miocene *Hystrix* discoveries in Turkey to two species: *H. primigenia* and *H. kayae*, with rare remains found at Kemiklitepe, Uşak, Türkiye, consisting of a hemimandible with a complete series of lower teeth, discovered by researchers from the Ege University Natural History Museum in the 1990s.

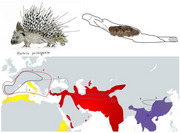

## INTRODUCTION

Porcupines (Hystricidae) constitute a distinctive and specialized family primarily composed of thorny and herbivore mammals. Extant porcupines are frequently targeted by medium and large predators such as lions, panthers, hyenas, wolves, and foxes (Mori *et al.* 2014; Viviano *et al.* 2020). These aposematic animals are well‐known for their scavenging diet, both in extant and the fossil record (Uldis 2012; Wieckowski *et al.* 2013, Mori *et al.* 2014; Coppola *et al.* 2020). Old World porcupines are classified within the family Hystricidae, comprising three distinct genera: namely *Trichys* and *Atherurus*, which belong to the subfamily Atherurinae, and *Hystrix*, which belongs to the subfamily Hystricinae (Happold 2013). The genus *Hystrix* comprises eight species, exhibiting the most diverse geographical distribution and is further categorized into three subgenera (Corbet & Jones 1965; Happold 2013): *Acanthion*, found in South and Southeast Asia; *Thecurus*, endemic to Southeast Asia; and *Hystrix*, with the widest geographical distribution from Africa to Eurasia (Amori *et al.* 2008; Şen & Purabrishemi 2010; Azzara *et al.* 2022). Although its fossil records are limited in many localities, this adaptable and widely distributed genus likely inhabited similar geography from the Late Miocene to recent times (Wang & Qiu 2002; Van Weers & Rook 2003; Şen & Purabrishemi 2010; Nishioka *et al.* 2011, 2020; Azzara *et al.* 2022). This conclusion is drawn from fossils attributed to *Hystrix* discovered in various Eurasian and African localities, dating as far back as the early Turolian and persisting until the Quaternary.

The earliest occurrence of the genus *Hystrix* is represented by *Hystrix parvae* (Kretzoi 1951) from Salmendingen (Germany) (MN9–11, NOW; MN6–17, Halaçlar *et al.* 2024), Kohfidisch (Austria) (MN6–11, NOW), and MN11 localities of Csakvar (Hungary) and Crevillente 2 (Spain) (Van Weers & Montoya 1996; Şen 2001a; Van Weers 2005; NOW 2023). Recent research by Halaçlar *et al.* (2024) has led to some uncertainty regarding the earliest findings of *Hystrix*. The study reports the discovery of a new *Hystrix* species, named *H. kayae*, and suggests that *H. parvae* and *H. kayae* are coeval, challenging previous assumptions about the earliest occurrence of the genus.

**Figure 1:**
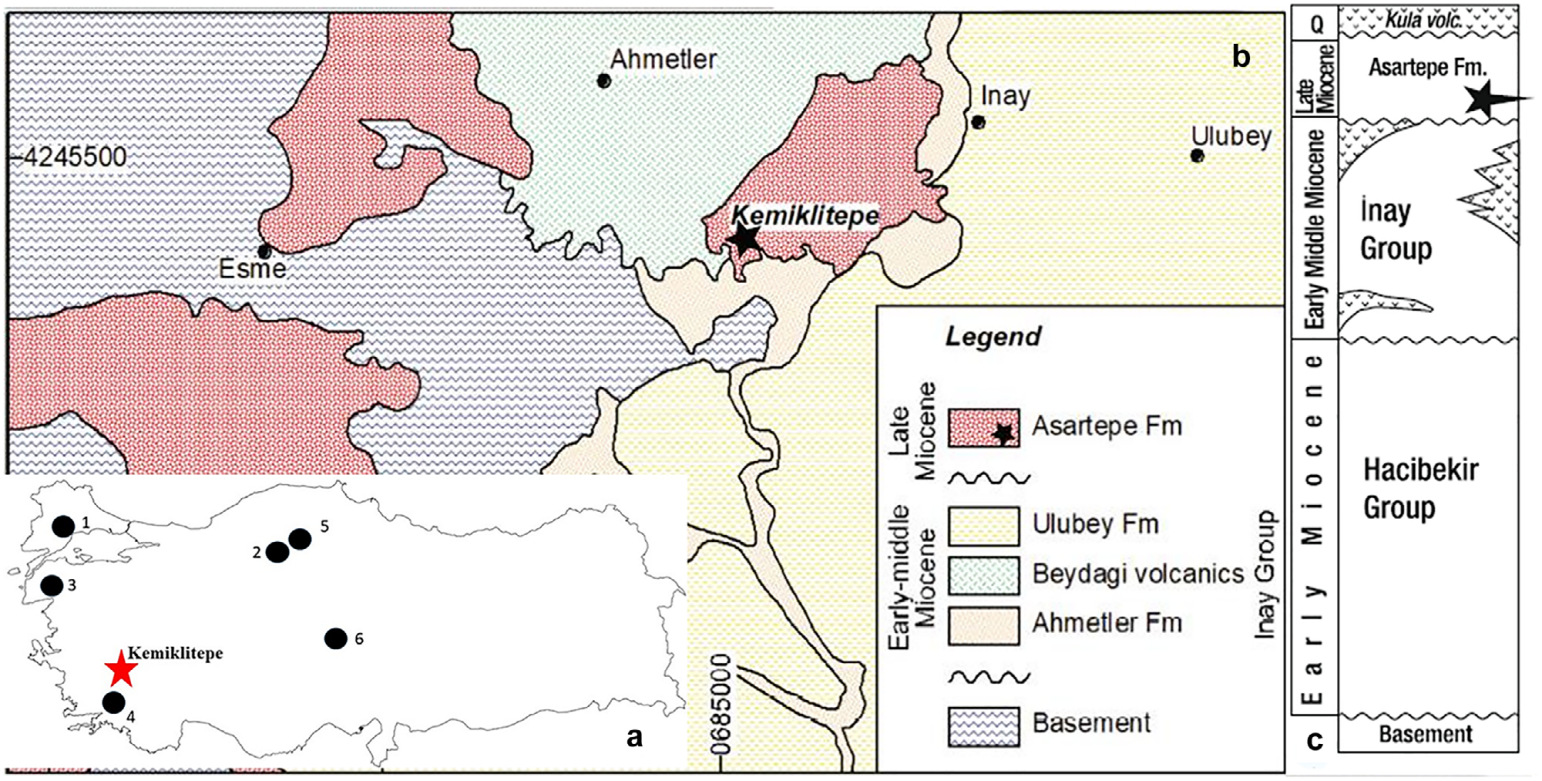
(a) Distribution of *Hystrix* species in Türkiye. 1, Bayırköy; 2, Çobanpınar; 3, Gülpınar; 4, Şerefköy; 5, Çorakyerler; 6, Taşkınpaşa, modified from Halaçlar *et al.* (2024). The red star is Kemiklitepe locality. (b) Geological map of the Kemiklitepe fossil locality and its surroundings (modified from Xafis *et al.* (2021) based on Karaoğlu *et al.* (2010)). (c) The stratigraphic position of the Kemiklitepe fossil locality revised by Seyitoğlu *et al.* (2009).

Three *Hystrix* species have been documented across seven localities from Late Miocene Anatolia (Fig. 1) (Atalay 1980; Ünay & Bruijn 1984; Şen 1994; Van Weers& Rook 2003; Pietro 2013; Halaçlar *et al.* 2024). The oldest among them, *H. kayae*, was identified in the Çorakyerler locality (Early Turolian, MN11). *H. primigenia* has been recorded in four Anatolian localities namely Bayırköy, Şerefköy, Kemiklitepe, and Gülpınar (Van Weers & Rook 2003; Halaçlar *et al.* 2024). The youngest species, *H. depereti*, was documented in Çobanpınar (MN13) (Van Weers & Rook 2003). Prieto (2013) reported an upper molar from Taşkınpaşa (MN11–13), designating the specimen as *Hystrix* sp. While *H. primigenia* is relatively common in Europe (Van Weers & Rook 2003; Şen & Purabrishemi 2010; NOW 2023), it appears to be absent from Asia (excluding Anatolia). *H. primigenia* is recorded in 30 Pliocene–Miocene localities in Eurasia (see Table S1, Supporting Information). The oldest record is in Kemiklitepe A‐B, while the youngest occurrences are in Kvabebi, Georgia and Raciszyn 1, Poland (Van Weers & Rook 2003; Şen & Purabrishemi 2010; Fejfar & Sabol 2022; NOW 2023). Şen (2001b) re‐described Perpignan porcupines and reallocated them to a new *Hystrix* species, named *H. depereti*. Subsequently, Van Wears and Rook (2003) provide a review of Turolian porcupines, reallocating certain *H. primigenia* findings to *H. depereti* based on size and enamel height. This study will further discuss these reallocations.

Şen (1994) conducted the initial study on Kemiklitepe *Hystrix*, describing two upper series and a mandibular fragment that included p4 and incisor fragments. In this study, we present the analysis of a mandible fragment discovered in the Late Miocene of Kemiklitepe‐A, Türkiye. These fragments constitute the first lower cheek teeth series of *H. primigenia* from Kemiklitepe. The identification of this fossil enhances our ability to compare the Kemiklitepe‐A specimen with those from various locations.

### Locality

The initial exploration of fossils at Kemiklitepe was conducted by Yalçınlar (1946). Subsequent studies of the fauna were carried out by Ozansoy (1969, 1957), Becker‐Platen *et al.* (1975), Tuna (1985), and Şen *et al.* (1994). The study by Şen *et al.* (1994) was part of an extensive monograph on the stratigraphy and paleoecology of the site, providing a detailed description of the new mammal collection. They established an estimated age of approximately 7.7–7.1 Ma for the fossil‐bearing formation, based on magnetostratigraphic correlation. This estimation was further corroborated by the presence of the typically Turolian fossil mammal assemblage.

Recent field investigations conducted by Seyitoğlu *et al.* (2009) and Karaoğlu *et al.* (2010) have determined that the fossiliferous sediments are part of the Asartepe Formation and are situated atop the Ulubey Formation, as depicted in Fig. 1. The Asartepe Formation is primarily composed of massive mudstones with fine‐grained conglomerate alternations supported by a matrix, indicative of subaerial deposition in a distal alluvial fan environment. The conglomerates contain pebbles to boulders derived from the underlying metamorphic basement rocks of the lacustrine Ulubey Formation and the volcanic Beydağı Formation, as reported by Seyitoğlu *et al.* (2009).

Based on the findings of Seyitoğlu *et al.* (2009), it is evident that the fossiliferous sediments within the Asartepe Formation were deposited after a period of erosion. This conclusion is supported by the examination of the valley floor and the contact zone between the Ulubey Formation and the Asartepe Formation and aligns with the findings of Ercan *et al.* (1978) and Seyitoğlu *et al.* (2009).

The previously identified fossiliferous horizons at Kemiklitepe comprise the upper and younger sites Kemiklitepe‐A (KTA), Kemiklitepe‐B (KTB), and Kemiklitepe‐C (KTC), along with the lower stratigraphic level of Kemiklitepe‐D (KTD), as documented by Şen *et al.* (1994). These two layers are vertically separated by a distance of 15 m. Faunal and magnetostratigraphic correlations point to a woodland/forest environment, with an estimated age of approximately 7.1 Ma for KTA, KTB, and KTC, and about 7.6 Ma for KTD, according to Şen *et al.* (1994). In a recent study, a new locality was identified in Kemiklitepe, situated 350 m northwest of the classic Kemiklitepe localities (Xafis *et al.* 2021). The new locality, named Kemiklitepe‐E (KTE), is positioned at the same stratigraphic level as the KTA–C localities (Xafis *et al.* 2021).

If there is no specific citation, given ages in brackets are sourced from the Neogene Old World database (NOW).

## MATERIALS AND METHODS

**Figure 2:**
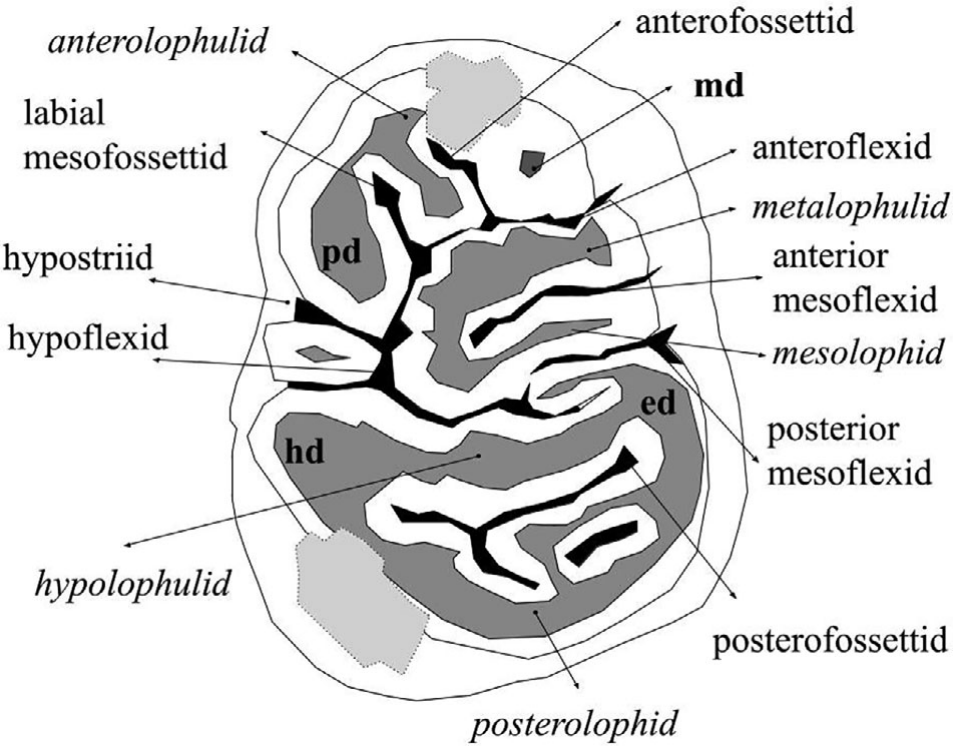
Anatomical nomenclature to describe lower cheek teeth structures of Hystricidae. Main cuspids are in bold and lophids in italics. pd, protoconid; ed, entoconid; hd, hypoconid; md, metaconid. The gray part represents the dentine. Light gray is broken parts. The dental nomenclature is after Lopatin *et al.* (2003) and Azzara *et al.* (2022).

The dental terminology utilized in this study adheres to the conventions established by Van Weers (1990), Şen (2001a), Lopatin *et al.* (2003), Van Weers and Rook (2003), and Azzara *et al.* (2022), Halaçlar *et al.* (2024) (Fig. 2). Azzara *et al.* (2022) present a comprehensive list of these terms in their literature, serving as the primary reference for this study (Azzara *et al.* 2022, online resource 2). Specifically, the term “system of wear classes,” was developed by Van Weers (1990) and Van Weers and Rook (2003) to classify the stages of tooth wear. Left‐side teeth are consistently designated as such in all visual representations, and reversed specimens are indicated by underlining the corresponding figures. All measurements were taken using an electronic caliper with a precision of 0.01 mm. The diagrams are generated using PAST 4.03 (Hammer *et al.* 2001)

Institutional abbreviations: EUNHM, Ege University Natural History Museum, Izmir; MNHN, Muséum national d'Histoire naturelle, Paris; UEKT = UEK = KT, Uşak, Esme, Kemiklitepe; PV, Paleontology Vertebrate; IPGM: Institut für Paläontologie und Historische Geologie, München; TSPI: Taganrog State Pedagogical Institute in Taganrog, Russia.

Terminologies: EH, the enamel height measured on the lingual side; EH/L, enamel height/length; L, length corresponding to the maximum values along the longitudinal tooth axe situated in the middle crown height; W, width corresponding to the maximum values along the transverse tooth axe in the middle crown height.

## SYSTEMATIC PALEONTOLOGY

**Order** Rodentia Bowdich, 1821

**Family** Hystricidae Fischer, 1817

**Genus** *Hystrix* Linnaeus, 1758

Type species: *Hystrix cristata* Linnaeus, 1758.

**Figure 3:**
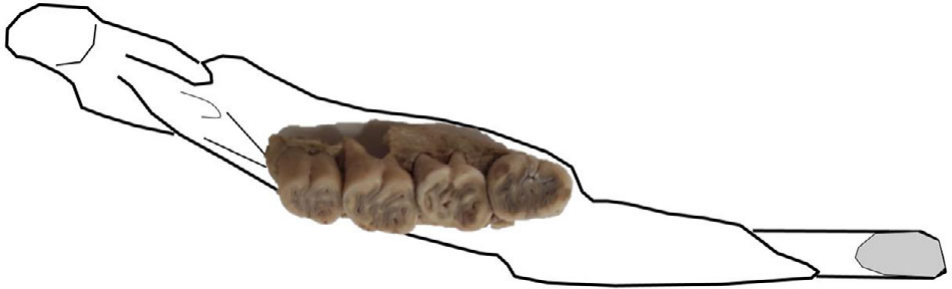
Partial left mandible with p4–m3 of *Hysterix primigenia* (PV‐UEKT‐A 88) from Kemiklitepe locality in occlusal view (scale bar equals 2 cm).

*Hystrix primigenia* Wagner, 1848 (Fig. 3)

**Figure 4:**
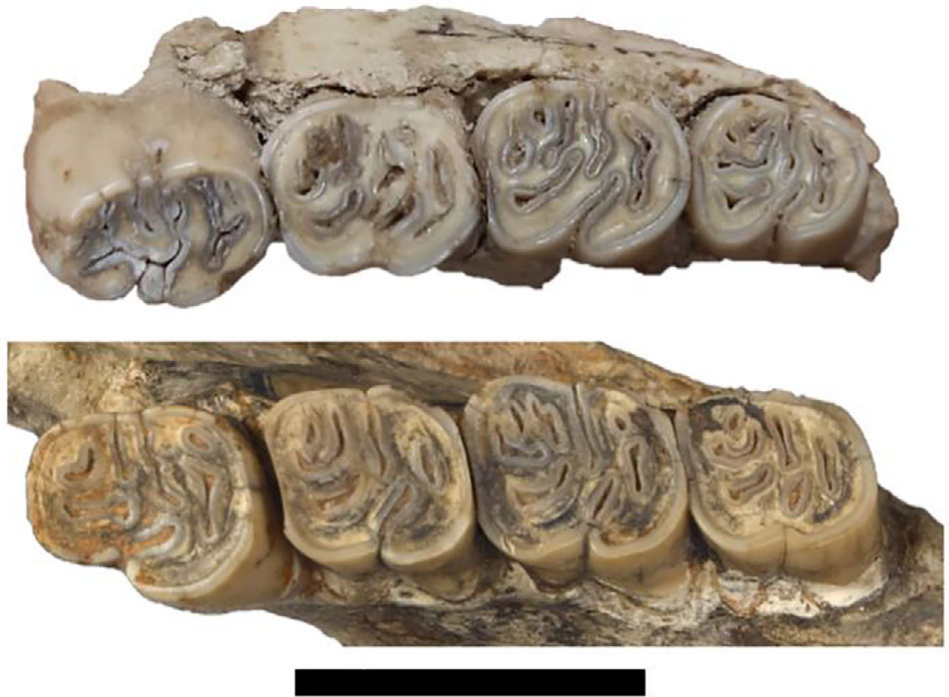
Kemiklitepe (PV‐UEKT‐A 88) (upper) and Pikermi (MNHN.F.PIK 3089) (lower) specimen (Gaudry 1862, figs 1,2; Şen 1999 figs 42.3,42.5). The scale bar equals 2 cm. MNHN.F.PIK 3089 taken from Muséum National d'Histoire naturelle, Paris (France) Collection: Paleontology (F). Credits for the photo: Elodie Lerat, 2018. http://coldb.mnhn.fr/catalognumber/mnhn/f/pik3089.

### Holotype

Left lower incisor fragment, IPGM ASII 146.

### Type locality

Pikermi, Greece.

### Geographical occurrence

Eurasia and North Africa.

### Chronological occurrence

Late Miocene to Early Pliocene.

### Referred material

PV‐UEKT‐A 88, left mandibular fragment with the left p4–m3, collection of the EUNHM. The mandibular bone is primarily preserved on the cheek teeth root.

### Locality

Kemiklitepe, Eşme/Uşak, Türkiye.

### Aged

Late Miocene, MN12 Mammal Neogene Zone.

### Measurements

See Table 1.

**Table 1:** Comparison of teeth measurements of *Hystrix primigenia* from Kemiklitepe, Pikermi, Bayırköy, Gülpınar, and Şerefköy and *H. depereti* from Perpignan

		Kemiklitepe	Pikermi	Bayırköy	Gülpınar	Şerefköy	Perpignan
			*H. primigenia*				*H. depereti*
p4	L	12.68	11.2 (4); 11–11.3	10.7	11.5	11.2	12.9 (5); 12.3–13.2
	W	10.32	9.9; 9.6–10.3	9.2	9.8	10	11; 10–11.8
	EH (E/L)	9.46 (0.74)	0.7; 0.41–0.95	(0.64)	(0.92)	(0.35)	0.89; 0.71–1.6
m1 (m1/2)	L	11.59	10.3 (9); 9–11.6	8.8–9.3	10.1–11.2	8.8–11.1	11.7 (10); 11.1–13.2
	W	9.81	8.8 (9); 8.4–9.4	8.2–8.3	9–9.8	9.0–10.0	11; 10–11.8
	EH (E/L)	4.7 (0.4)	0.54 (7); 0.39–0.72	(0.5–0.63)	(0.55–0.59)	(0.23–0.28)	0.78; 0.53–1.1
m2	L	11.77					
	W	9.95					
	EH (E/L)	6.67 (0.67)					
m3	L	10.32	9.7 (3); 9.4–9.8	9.6		10.4	10 (5); 9.6–10.5
	W	8.7	8.6; 8.4–9.4	7.6		9.9	8.7 (5); 8.4–9
	EH (E/L)	8.21 (0.8)	0.54; 0.39–0.72	(0.5)		(0.29)	0.82 (4); 0.55–1.01
p4–m3		45.54	41.6 (3); 39.4–43.4	39.8		41.2	47.5

For multiple measured specimens, the figure includes the mean value, the number of specimens, and the range. Data curated from Van Weers and Rook (2003) and Şen (2001a).

### Description

The teeth are well‐preserved in the left hemimandible fragment PV‐UEKT‐A 88 (Figs 3, 4, Supporting Information 2), while the mandibular bone is preserved primarily around the roots. Four roots of p4 are visible, and three of them are damaged. In the occlusal view, the lower cheek teeth exhibit a labial twist from posterior to anterior (i.e. the occlusal face of m3 bends lingually while p4 bends labially). In the lateral view, the connection line between the mandibular bone and teeth rises anteroposteriorly resulting in higher p4 hypsodonty than in molars. The anterior part of the molars is significantly larger than the posterior, while p4 displays the opposite pattern. The occlusal surface of p4 corresponds to wear class O3. The condition of m3 (S1) suggests that the Kemiklitepe specimen belongs to age class V, according to Van Weers's (1990) classification. This information indicates that the Kemiklitepe specimen represents a subadult individual.

**Figure 5:**
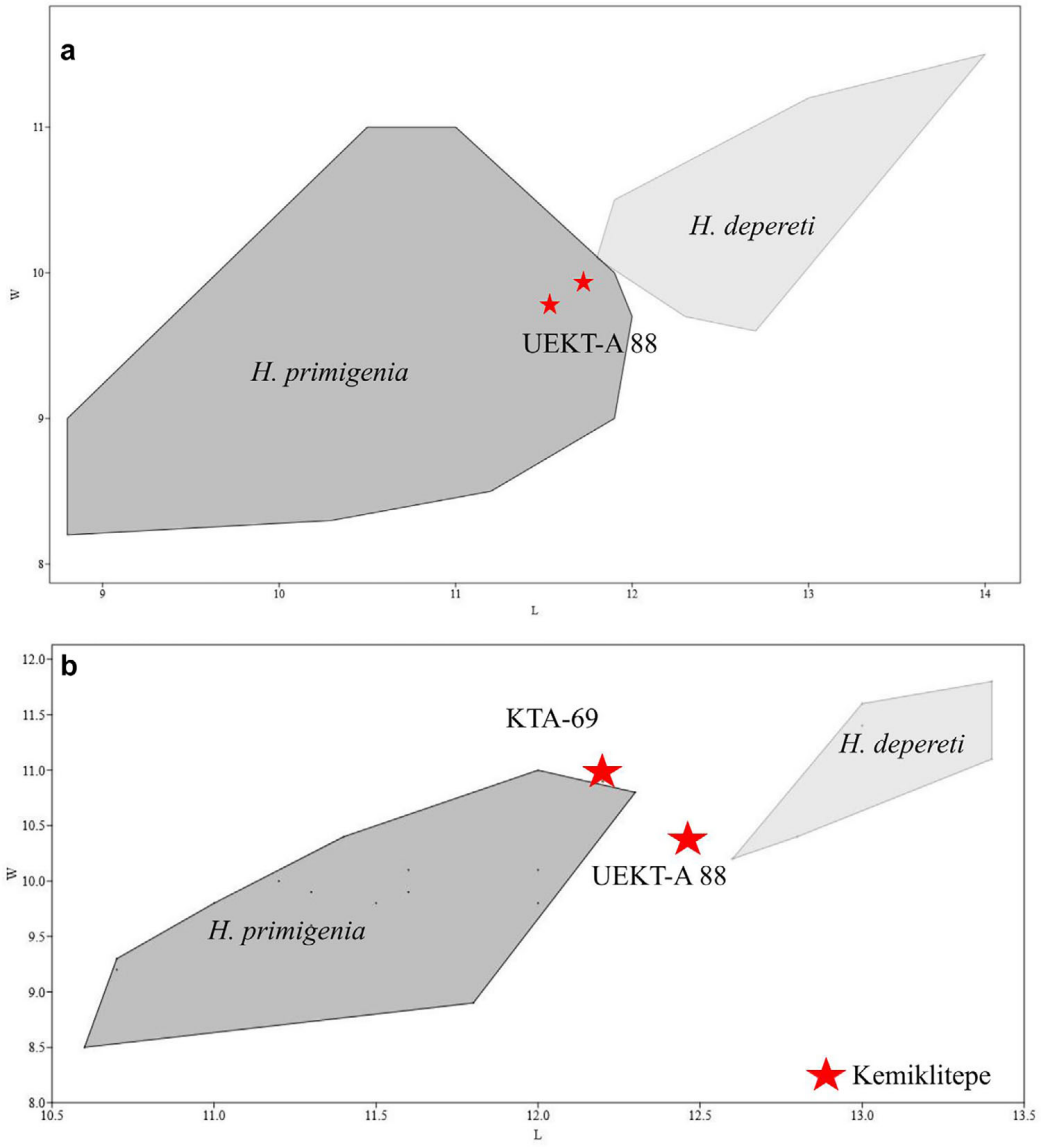
Length (L)–width (W) diagram of (a) m1/2 (top) and (b) p4 (bottom) of *Hystrix primigenia* and *H. depereti* from Europe and Asia Minor. Data source: Şen (1999, 2001a, 2001b), Lopatin *et al.* (2003), Van Weers and Rook (2003), and Şen and Purabrishemi (2010).

The occlusal surface of p4 exhibits a water drop shape from anterior to posterior, with all cusps fully erupted. In the occlusal view, the hypoflexid is narrow and aligned parallel to the main axis of the p4. The hypostriid crosses vertically on the labial wall of the crown, terminating 4.65 mm above the root line. Four lingual folds are visible in the occlusal view. The anterofossettid separates the metaconid from the anterolophulid and is connected to both the anteroflexid and the hypoflexid. The anteroflexid labiolingually reaches to hypoflexid and throughout this stretch and it connects with the anterofossettid and the labial mesofossettid. The anterior mesoflexid opens on the lingual side and closes on the labial side due to the connection between the metalophulid and mesolophid. The metalophulid and mesolophid are connected, forming a “U” shape lingually. The labial mesofossettid nearly reaches the mesial border of the tooth, separating the protoconid from the anterolophulid. The posterior mesoflexid reaches the hypoflexid, dividing the occlusal surface of p4 into two parts. Lingually, the posterior mesostriid and anterior mesostriid are visible, while the anterostriid is not well visible. The posterofossettid does not open on the border of the tooth and forms two enamel islets in the posterolophid. A robust labiolingual connection between the hypoconid and entoconid is established via the hypolophulid. Distally, a similar connection between the hypoconid and entoconid is formed by the posterolophid in a “U” shape. Between the protoconid and hypoconid, a small additional/accessory closed lophulid is present, restricted by the hypoflexid and labial mesofossettid. The less worn metaconid is not as prominent as the protoconid.

The occlusal surfaces of m1 and m2 are rectangular while the m3 is trapezoidal. In the occlusal view of the molar series, the anterolingual corner is more robust and less unworn than other corners.

The m1 has the lowest enamel height (EH) among the lower cheek teeth and is slightly smaller than m2 (Figs 3, 4, and Table 1). The tooth is worn nearly to the hypostriid, with the hypostriid crossing vertically on the labial wall of the crown and terminating 4.28 mm above the root line. The hypoflexid has transformed into an islet, contributing to the seven enamel islets on the occlusal surface of the m1. The posterofossettid retains a narrow ellipse shape. There is no connection between the posterior mesoflexid and hypoflexid, and they both form islets. The labial mesofossettid is anterolingually concave. The anterofossettid consists of two islets, and the anterior mesoflexid runs labiolingually, unable to reach the labial mesofossettid. In the anterior part, the metaconid is considerably stronger than the protoconid, while in the posterior part, the hypoconid is stronger than the entoconid.

Apart from differences in size and configuration of the posterior mesoflexid and hypoflexid, the occlusal outline and pattern of m2 closely resemble those of m1. While the posterior mesoflexid and hypoflexid in m2 are not islets and instead fold, the posterior mesoflexid aligns linguolabially, while the hypoflexid aligns posterolingually. The labial mesofossettid and posterofossettid of m2 exhibit slightly more prominent than m1. The hypostriid in m1 and m2 terminates 4.45 and 4.33 mm above the root line, respectively.

The occlusal outline and size of m3 differ slightly from the other molars, although the occlusal outline itself is similar. A notable distinction in the occlusal pattern lies in the connection between anterofossettid and anterior mesoflexid. The anterior part is twice the size of the posterior part, resulting in m3 adopting a teardrop shape. The hypostriid terminates 3.98 mm above the root line.

## COMPARISONS AND DISCUSSIONS

Distinguishing characteristics of *Hystrix* species in various studies include the occlusal pattern, occlusal outline, enamel height, and cheek teeth size. (Van Weers 1990; Şen 2001a, 2001b; Lopatin *et al.* 2003; Van Weers & Rook 2003; Şen & Purabrishemi 2010; Flynn & Wu 2017; Fejfar & Sabol 2022; Azzarà *et al.* 2022; Halaçlar *et al.* 2024). Although the occlusal pattern of *Hystrix* species generally exhibits a degree of conservatism, it remains an informative feature. In systematic studies, the occlusal outline frequently serves as a pivotal criterion, with size being the primary discriminant for taxa differentiation (Van Weers & Rook 2003; Şen & Purabrishemi 2010; Fejfar & Sabol 2022; Halaçlar *et al.* 2024). Şen (2001a) and Van Weers and Rook (2003) have noted a significant transition in hypsodonty among *Hystrix* species during the middle Turolian period. Notably, later Miocene species exhibit a heightened degree of hypsodonty, as exemplified by *H. depereti* in the latest Miocene.

In this part, we will perform a comparison using these four key characters: the occlusal pattern, occlusal outline, enamel height, and cheek teeth size. Considering the age of Kemiklitepe, we primarily focus on the middle Turolian Anatolian porcupines, specifically *H. primigenia* and *H. depereti*. However, initially, we will exclude other Eurasian porcupines from our analysis.

The oldest *Hystrix* species are *H. kayae* and *H. parvae*. *H. parvae*, being a small porcupine, can be readily distinguished from the Kemiklitepe specimen by its size and occlusal pattern (Fig. 3 and Table 1; Van Weers & Montoya 1996, plate 1). Conversely, *H. kayae* shares a similar size with the Kemiklitepe sample, but it exhibits a wholly distinct occlusal outline and pattern (Şen 1999, fig. 1; Halaçlar *et al.* 2024, fig. 5). It is important to note that direct comparisons between the two are not feasible here, primarily due to the difference in sample composition—*H. kayae* consists of maxillary samples, whereas our sample is hemimandible. Similar to *H. kayae, H. aryanensis* shares a comparable size with the Kemiklitepe specimen and is primarily composed of maxillary samples. However, it is worth noting that its occlusal pattern and outline exhibit subtle distinctions when compared to the Kemiklitepe porcupine (Şen 1999, fig. 1; Halaçlar *et al.* 2024, fig. 5).

The Pikermi *H. primigenia* specimen displays a pattern of increased wear from m1 to m3, resulting in a decrease in EH (enamel height). In contrast, the Kemiklitepe specimen demonstrates the opposite trend, with EH decreasing from m3 to m1. The Pikermi specimen records its lowest EH at m3, whereas for the Kemiklitepe specimen, the lowest EH is observed at m1. In both the Kemiklitepe and Pikermi specimens, the lower cheek teeth exhibit linguolabial twisting from m3 to p4. Additionally, in both specimens, there is a slight reduction in size from m2 to m1, with m1 being slightly smaller than m2. Both specimens share similar occlusal outlines for all teeth, except for m3. In the Pikermi specimen, m3 exhibits a rectangular shape, whereas in the Kemiklitepe specimen, it takes on a teardrop shape. Although the occlusal pattern in m1 and m2 is notably similar, discernible differences become apparent in p4 and m3. It is important to consider that these distinctions can be attributed to wear levels and the intensity of occlusal pattern variations. According to Şen (1994), the upper series of the Kemiklitepe specimen has quite a similar size, occlusal outline, and pattern to the Pikermi specimen while P4 is slightly larger than in the Pikermi specimen (Şen 1994, fig. 1; Van Weers & Rook 2003, fig. 6A).

The Kemiklitepe porcupine exhibits smaller teeth size compared to *H. depereti* from Perpignan (Fig. 4 and Table 1). Notably, the most distinctive feature of *H. depereti* is its higher degree of hypsodonty when compared to other Late Miocene European *Hystrix* species, as documented by Şen (2001a) and Van Weers and Rook (2003). In contrast, the hypsodonty of the Kemiklitepe specimen is more akin to *H. primigenia*, although notably shorter than *H. depereti* (Table 1). Regarding the occlusal outlines of the Perpignan and Kemiklitepe porcupines, they exhibit similarities, except for m3 (Fig. 3; Şen 2001a, fig. 4). In the Kemiklitepe specimen, m3 takes on a slightly teardrop shape, whereas m3 of the Perpignan porcupine is triangular (Fig. 3; Şen 2001a, fig. 4). This renders the Kemiklitepe specimen more akin to the Pikermi specimen. The p4 of the Kemiklitepe specimen is notably larger than that of the Pikermi specimen but still falls short of the size observed in the Perpignan specimen (Fig. 4). While there are slight variations in the occlusal patterns among the three samples, these differences can be attributed to varying levels of wear.

**Figure 6:**
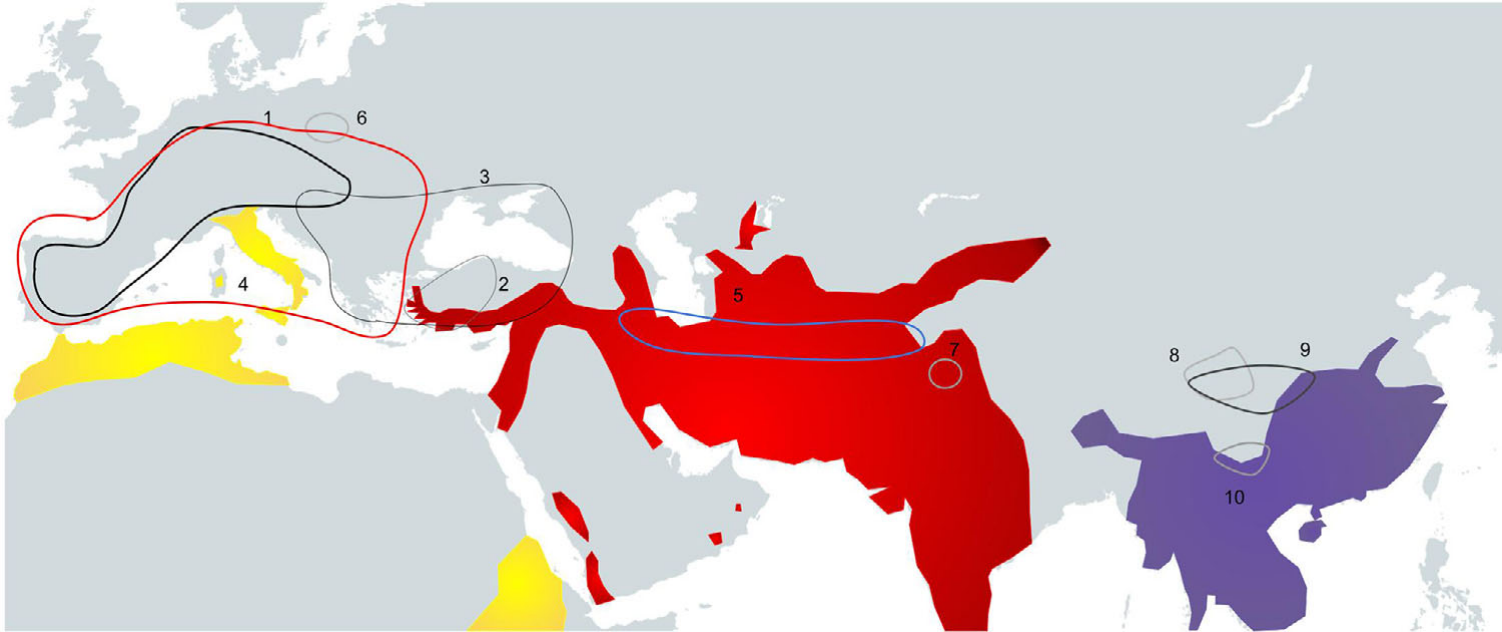
Paleogeographical distribution of Eurasian Late Miocene porcupines and some extant species. Extant species: Yellow—*Hysterix cristata*; red—*H. indica*; purple—*H. brachyura*. Fossil species: (1)* Hystrix parvae*, (2) *H. kayae*, (3) *H. primigenia*, (4) *H. depereti*, (5) *H. aryanensis*, (6) *H. velunensis*, (7) *H. primigenia?* (*sivalensis*), (8) *H. brevirostra*, (9) *H. gansuensis*, and (10) *H. lufengensis*. Extant species geography is modified from Mori *et al.* (2019).

Based on the data presented in Fig. 5, the molar size of the Kemiklitepe specimen falls within a size range similar to that of *H. primigenia*, although it is slightly smaller than the range observed in *H. depereti*. In general, it closely aligns with the size range of Hadzhidimovo *H. primigenia*. In terms of p4 width, the Kemiklitepe specimen falls within the range of *H. primigenia*, albeit slightly longer than the measurements of all *H. primigenia* specimens. However, it remains smaller in size when compared to all Perpignan *H. depereti* specimens. Fig. 6

Additional Late Miocene Anatolian *Hystrix* samples have been collected from various localities, including Çorakyerler, Çobanpinar, Bayırköy, Şerefköy, Gülpınar, and Taşkınpaşa in Türkiye (Fig. 1 and Table 1) (Ünay & Bruijn 1984; Şen 1994; Atalay 1980; Van Weers & Rook 2003; Prieto 2013; Halaçlar *et al.* 2024).

Taşkinpaşa: An isolated molar sample from Taşkinpaşa exhibits an occlusal pattern and outline noticeably similar to the upper M1 of the Kemiklitepe specimen. However, it is worth noting that the Taşkinpaşa sample is slightly longer and narrower in dimensions when compared to the Kemiklitepe specimen (Şen 1994, fig. I and table 1; Pietro 2013, fig. 1).

Çobanpinar: Van Weers and Rook's (2003) analysis attributes the Çobanpinar specimens to *H. depereti* based on their EH and larger size. Interestingly the upper dentition of the Kemiklitepe and Çobanpinar samples are closely matched in size and exhibit similar occlusal patterns, with the exception of P4, as highlighted in Şen (1994, table I and fig. 1), and Van Weers and Rook (2003, table 4 and fig. 6B). This suggests that the Perpignan *H. depereti* differs more significantly from the Kemiklitepe sample than the Çobanpinar sample does. In this context, our preference is to classify it as *H. primigenia*, although it undeniably warrants further study and examination.

According to the classifications provided by Van Weers and Rook (2003), the specimens from Bayırköy, Şerefköy, and Gülpınar are assigned to *H. primigenia*. We adhere to their categorization, as it is based on size, EH, and occlusal outline. Additionally, the porcupine specimens from Çorakyerler have been described as a new *Hystrix* species named *H. kayae*, with their distinctions from *H. primigenia* comprehensively elucidated in the study by Halaçlar *et al.* (2024).

### *H. primigenia* in Eurasia during Late Miocene (in time and space)

Over the past two decades, several comprehensive studies have delved into the *Hystrix* species of the Late Miocene period in Eurasia and North Africa (Van Weers & Rook 2003; Lopatin *et al.* 2003; Şen & Purabrishemi 2010; Kovachev 2012; Halaçlar *et al.* 2024). Van Weers and Rook (2003) examined the dispersal patterns of *Hystrix* in the Old World, discussing four prominent species: *H. parvae, H. aryanensis, H. primigenia*, and *H. depereti*, with a primary aim to reassign some *H. primigenia* samples to *H. depereti*. Subsequently, Lopatin *et al.* (2003) restricted the geographic range of *H. depereti* to Perpignan, the type locality of *H. depereti*. Şen and Purabrishemi (2010) made a significant contribution by introducing an additional finding of *H. aryanensis*, and they concluded that “the spatial distribution of *H. primigenia* is restricted to southeastern Europe and Asia Minor during the middle‐late Turolian.” Kovachev (2012) focused on cranial and postcranial materials of *H. primigenia* from Hadzhidimovo, Bulgaria, listing *H. primigenia* localities by country and agreeing with Lopatin *et al.* (2003) about *H. depereti* representing only Perpignan.

### *H. primigenia* versus *H. depereti*

Until Şen (2001a) established the new species *H. depereti, H. primigenia* was predominantly known as the sole *Hystrix* species in Europe and Anatolia during the Late Miocene. Following this significant study, Van Weers and Rook (2003) reclassified most *H. primigenia* as *H. depereti* based on enamel height and size of cheek teeth (Table S1, Supporting Information). Şen and Purabrishemi (2010) largely confirmed the results of Van Weers and Rook (2003) (Table S1, Supporting Information).

- The EH of *H. depereti* is higher than Late Miocene porcupines (*H. parvae, H. kayae, H. aryanensis, H. primigenia, H. gansuensis, H. lufengensis, H. brevirostra*, and *H. paukensis*) and smaller than that of Plio‐Pleistocene porcupines (Wang & Qiu 2002; Van Weers & Rook 2003; Wang & Qi 2005; Şen & Purabrishemi 2010; Nishioka *et al.* 2011; Wang & Qiu 2020; Halaçlar *et al.* 2024).
- The last molar of *H. depereti* is reduced compared to those of *H. primigenia, H. kayae*, and *H. aryanensis*.
- There are no significant differences in occlusal outline and pattern between *H. primigenia* and *H. depereti*.

Until more detailed comparative studies and additional data become available, the distinction between *H. depereti* and *H. primigenia* cannot be drawn with greater clarity.

## PALEOGEOGRAPHY OF *H. PRIMIGENIA*

In this section, we provide information about the presence of *H. primigenia* in Eurasia by country, and in addition, we include *H. depereti* localities.

### Pakistan

In the Late Miocene of Pakistan, the *Hystrix* genus is represented by a single species found in the Siwalik, Dhok Pathan Formation—*H. sivalensis*, Lydekker (1878). Van Weers and Rook (2003) reclassified it as *H. primigenia*. However, Şen and Purabrishemi (2010) designated it as *Hystrix* sp., citing the limited available material, consisting of a right mandible fragment with m1 and m2, and nothing that that the hypsodonty of the lower cheek teeth lacks the distinctiveness seen in the upper cheek teeth (Şen & Purabrishemi 2010).

### China

Three *Hystrix* species, namely *H. lufengensis, H. gansuensis*, and *H. brevirostra*, are endemic to the Late Miocene of China, as documented by several studies (Wang & Qiu 2002; Van Weers 2004; Wang & Qi 2005; Flynn & Wu 2017; Wang & Qiu 2020). Initially, Van Weers (2004) initially attributed porcupine remains at the Lufengpithecus site to *H. primigenia*. However, Wang and Qi (2005) established a new species, *H. lufengensis*, following extensive analysis. *H. lufengensis* features distinctive characteristics, including squarer cheek teeth, a shorter p4–m3 series, and a less reduced m3 compared to *H. primigenia*. *H. gansuensis* differs from *H. primigenia* with its high‐crowned and smaller cheek teeth (Wang & Qiu 2002). On the other hand, *H. brevirostra* is characterized by higher cheek teeth, a shorter rostrum, a lower horizontal ramus of the mandible, and a shorter and deeper concave mandible diastema compared to *H. primigenia* (Wang & Qiu 2020).

### Russia

Lopatin *et al.* (2003) reported the first *H. primigenia* record from the late Turolian (MN13) locality Morskaya 2, Russia. The material, an isolated P4 (TSPI M‐2/86‐1) (Lopatin *et al.* 2003, fig. 3), is larger than the Kemiklitepe specimen (KT‐1) (Şen 1994, table 1 and fig. 1).

### North Macedonia

Spassov *et al.* (2018) documented four middle‐late Turolian localities for *H. primigenia* in North Macedonia, namely Choloshevski Dol, Kiro Kucuk, Umen Dol, and Vesje. Kiro Kucuk is represented by a mandible, Umen Dol by a maxilla, and Vesje by a partial skull, as outlined by Spassov *et al.* (2018). The porcupine from Choloshevski Dol was previously reported by Garevski (1956), and a skull fragment with upper molars was described by him.

### Spain

According to NOW, Spain has one record of *H. primigenia* during the Late Miocene in Villastar (Teruel) (Mein *et al.* 1990). In the Pliocene, there are two records, Layna, Villarroya (Agusti *et al.* 1987), and Villalba Alta 1 (Adrover 1986).

Alcala and Montoya (1998) initially assigned porcupine materials from Las Casiones to *H. primigenia* later reclassified by Van Weers and Rook (2003) as *H. depereti* based on enamel height and size. In this study, we observe a close resemblance in the occlusal outline of the m3 from Las Casiones to those characteristics of *H. depereti*. Following the methodology and conclusions of Van Weers and Rook (2003), we classify the porcupine materials from Las Casiones as *H. depereti*. Similarly, the specimens from Villastar have been reexamined and subsequently reclassified as *H. depereti* by Van Weers and Rook (2003).

### Italy

In Late Miocene Italy, three *Hystrix* records exist: Brisighella (Monticino), Baccinello V3, and Piedmont as documented by Hurzeler and Engesser (1976), Masini and Rook (1993), and Colombero *et al.* (2015), respectively. Masini and Rook (1993) initially assigned the Brisighella porcupine specimen to *H. primigenia* but later classified material from Baccinello V3 as *Hystrix* sp. Subsequently, Van Weers and Rook (2003) reclassified Brisighella porcupine as *H. depereti*. Colombero *et al.* (2015) studied the porcupine materials from Verduno, Piedmont (MN13), including an isolated upper molar and a radius fragment, finding the enamel height consistent with *H. depereti*.

### Albania

Festani *et al.* (1997) studied porcupine materials from the Late Miocene–early Pliocene locality of Shahinova, Albania, assigning them to *H. primigenia*. This specimen exhibits a reduced m3, with no information on its hypsodonty in the article. Further study may enhance its comparison with *H. depereti*.

### Moldova

The porcupine discovered in the Late Miocene locality of Taraklia (Moldova) was initially described as *H. bessarabica* by Riabinin (1929) and was later reclassified to *H. primigenia* by Van Weers and Rook (2003).

### France

A reassessment of the Perpignan material led to the determination that the specimens unequivocally pertain to a new *Hystrix* species, identified as *H. depereti* (Şen 2001b). Comparative reexamination of tooth size and crown height between Pikermi and Perpignan porcupine highlights that *H. depereti* surpasses *H. primigenia* in both size and crown height (Şen 2001b; Van Weers & Rook 2003).

### Hungary

The initial report of *H*. cf. *primigenia* findings from Polgardi (MN13), Hungary, by Kormos (1911) was later scrutinized by Van Weers and Rook (2003), who concluded that they could not be definitively attributed to any particular species due to the mixed nature of the collection.

### Bulgaria

Five *H. primigenia* findings are reported in Bulgaria: Kalimanci, Strumyani, Hadzhidimovo, Azmaka 6, and Staniantsi (Şen & Korachev 1987; Geraads *et al.* 2011; Kovachev 2012; Spassov *et al.* 2012; Bullmann *et al.* 2013). The Strumyani materials consisting of cranial and postcranial elements were attributed to *H. primigenia* by Geraads *et al.* (2011). Kovachev (2012) studied an almost completed *H. primigenia* skeleton from Hadzhidimovo (MN12), providing a rare opportunity to compare potential postcranial *Hystrix* findings. Bullmann *et al.* (2013) reported another *H. primigenia* finding (p4–m2) from Staniantsi site (MN13–14).

### Greece

Recent palaeomagnetic data suggest an age of 7.33–7.29 Ma for Pikermi, the type locality of *H. primigenia*, that confirms the correlation with the upper part of MN12 (Böhme *et al.* 2017; Koufos 2023). In the 1960s, two *H. primigenia* findings from two Turolian Greek localities, Alifakas (7.6–7.1 Ma) and Halmyropotamos (8.9–5.3 Ma) were reported by Melentis and Schneiders (1966) and Melentis (1967). Bonis *et al.* (1992) identified three *H. primigenia* specimens from Dytiko‐3 (7.1–5.3 Ma), Chomateres (7.6–7.1 Ma), and Halmyropotamos in the Late Miocene Greece as detailed by Koufos *et al.* (2011). Lazaridis *et al.* (2018) introduced *H. primigenia* finding from Kryopigi (8.9–5.3 Ma).

In Samos (7.1–5.3 Ma), Solounias (1981) initially attributed findings to *H. primigenia*, which include a skull and a mandible fragment with the incisor and a portion of the m3. However, Van Weers and Rook (2003) reassigned it to *H. depereti* based on characteristics such as high hypsodonty, large‐sized cheek teeth, and a reduced last molar. A notable observation is that *H. depereti* from Perpignan and *H. primigenia* from Pikermi exhibit square‐shaped upper molars (Şen 2001a, fig. 2; Van Weers & Rook 2003, fig. 6). The Samos specimen's occlusal outline is more similar to more rectangular Çorakyerler *H. kayae* (Halaçlar *et al.* 2024). A distinguishing feature of *H. kayae* is the presence of an extra anterolingual groove on P4, while the Samos specimen exhibits DP4. Based on these observations, we propose that the Samos specimen bears a closer resemblance to *H. kayae* than to either *H. depereti* or *H. primigenia*. Thus, we designate the Samos specimen as *H*. cf. *kayae*.

### Türkiye

In the comparison and discussions part, we provided a detailed analysis of *Hystrix* findings in Türkiye. In summary, we allocated all Turkish findings to *H. primigeni*a, excluding the Çorakyerler porcupine, rejecting the presence of *H. depereti* in Türkiye and confining its distribution to Europe and North Africa.

During the Pliocene, *H. primigenia* and *H. depereti* are documented across three countries. Fejfar and Sabol (2022) reported *H*. cf. *depereti* from the early Pliocene locality Ivanovce (fissure 6513) in Slovakia. In Menacer, Algeria (Africa), Arambourg (1959) initially reported *H. primigenia*, which was later reassigned to *H. depereti* by Van Weers and Rook (2003). Georgia's sole *H. primigenia* records are from Kvabebi (MN16, NOW) (Vekua 1972). In Weze, Poland, fossils initially identified as *H. primigenia* by Sulimski (1960) were reclassified as *H. depereti* by Van Weers and Rook (2003). In the most recent study, a new species, *H. velunensis*, was introduced by Czernielewski (2023).

**Figure 7:**
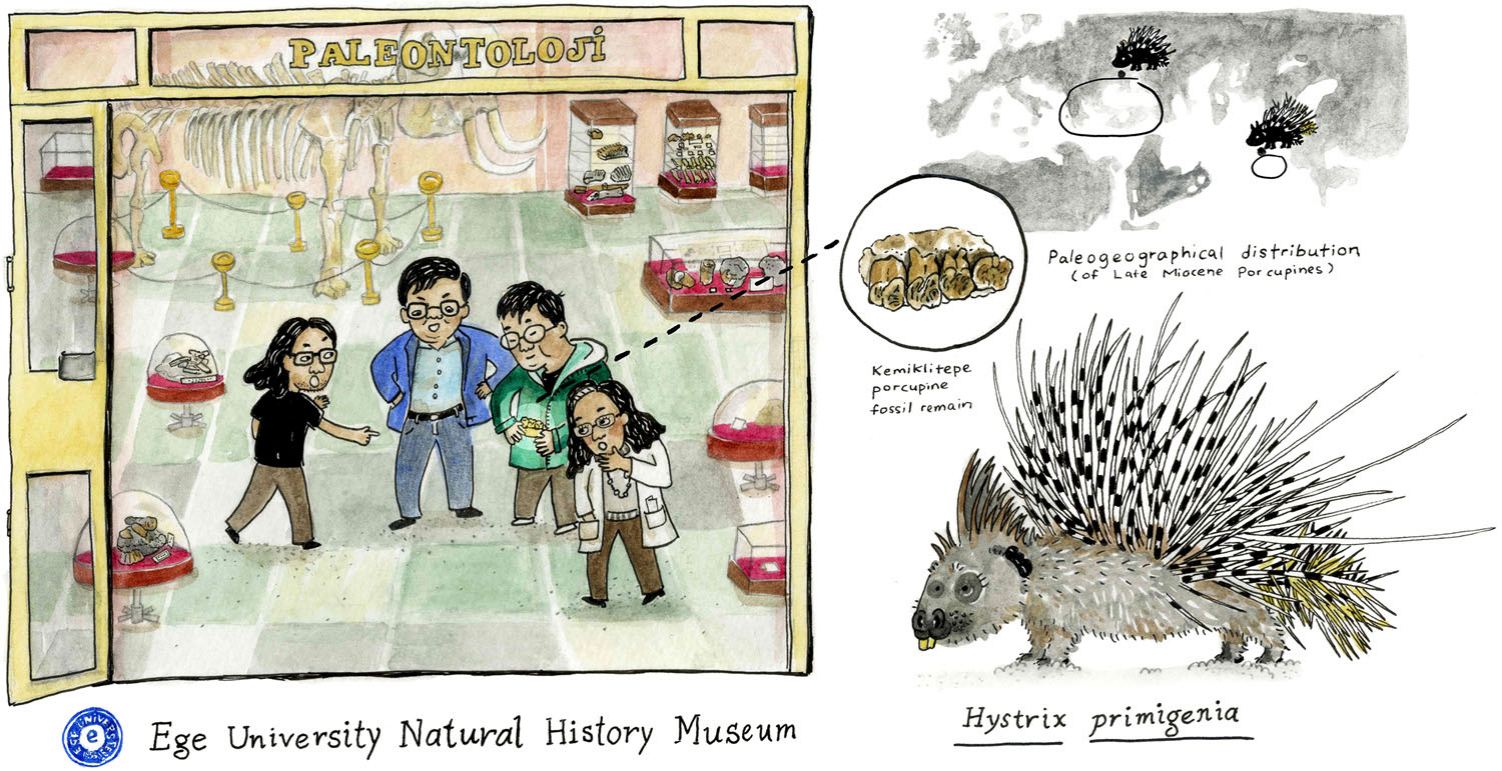
The illustration depicts interactions between museum scientists and visitors, illustrating the process of creating paleogeographic maps based on a single fossil finding. Artwork by NOvia Shin.

In Fig. 7, a visual representation unfolds, capturing the dynamic interactions between museum scientists and visitors. This illustrative scene encapsulates the collaborative efforts involved in the detailed process of drawing paleogeographic maps. The focus centers on the utilization of a singular fossil finding, showcasing the intriguing intersection of scientific inquiry and public engagement within the museum setting.

## CONCLUSION

This study supports the findings of Şen (1994), who examined an upper cheek tooth series alongside a mandible fragment with a lower incisor and p4. Based on the examination of a hemimandible fragment preserved at EUNHM, the porcupine specimens from Kemiklitepe have been identified as *H. primigenia*. Our paleobiogeographical analysis reveals that *H. depereti* was absent in Late Miocene Türkiye, highlighting a broader dispersal range for *H. primigenia* across Eurasia compared to *H. depereti*. Notably, both species were not present in Asia, with the exception of Anatolia for *H. primigenia*, during the Late Miocene. Furthermore, our findings suggest the presence of *H. kayae* in Samos, Greece, indicating that Greece hosted a third *Hystrix* species in the Late Miocene.

## Supporting information

Supplementary Materials

**Table S1** Original studies

**Supporting Information 2** 3D scans of *Hystrix primigenia* specimen (pptx and ply document)

## References

[inz212820-bib-0001] Adrover R (1986). Nuevas Faunas de Roedores en el Mio‐Plioceno Continental de la Region de Teruel (España): Interes Bioestratigrafico y Paleoecologico. Instituto de Estudios Turolenses de la Exca, Diputación Provincial de Teruel, Spain.

[inz212820-bib-0002] Agusti J , Arbiol S , Martin Suarez E (1987). Roedores y Lagomorfos (Mammalia) del Pleistoceno inferior de Venta Micena (depresion Guadix‐Baza). Paleontologia y Evolucion, Memoria Especial 1, 95–107.

[inz212820-bib-0003] Alcalá L , Montoya P (1998). *Hystrix primigenia* (Wagner, 1848) (Rodentia, Mammalia) del Mioceno Superior (MN13) de Las Casiones (Fosa de Teruel, España). Revista Espanola de Paleontologia 13, 139–147.

[inz212820-bib-0080] Amori G , Contoli L , Nappi A (2008). Mammalia II: Erinaceomorpha, Soricomorpha, Lagomorpha, Rodentia. Fauna d'Italia, vol. 40. Il Sole 24 Ore. Edizioni Calderini, Bologna.

[inz212820-bib-0005] Arambourg C (1959). Vertébrés continenteaux du Miocène supérieur de l'Afrique du Nord. Publications du Service de la Carte Geologique de l'Algerie. Paleontologie 4, 101–161

[inz212820-bib-0006] Atalay Z (1980). Stratigraphy of continental Neogene in the region of Mulgla‐Yatagan, Turkey. Bulletin of the Geological Society of Turkey 23, 93–99.

[inz212820-bib-0007] Azzarà B , Cherin M , Adams J *et al*. (2022). The thorny issue of African porcupines: A new mandible of *Hystrix makapanensis* from Olduvai Gorge (Tanzania) and rediagnosis of the species. Journal of Mammalian Evolution 29, 447–474. 35079214 10.1007/s10914-021-09588-z PMC8776392

[inz212820-bib-0083] Becker‐Platen JD , Sickenberg O , Tobien (1975). Vertebraten‐Lokalfaunen der Türkei und ihre Altersstellung. In: Sickenberg O , ed. Die Gliederung des Höheren Jungtertiärs und Altquartärs in der Türkei nach Vertebraten und ihre Bedeutung für die Internationale Neogen‐Stratigraphie (Känozoikum und Braunkohlen der Türkei. 17.). Schweizerbart Science Publishers, Stuttgart, pp. 47–100.

[inz212820-bib-0010] Bullmann L , Böhme M , Prieto J , Spassov N (2013). *Hystrix* primigenia from the Staniantsi Locality (Miocene‐Pliocene Transition) in Western Bulgaria. In: Book of Abstracts of the RCMNS. Istanbul Technical University, Istanbul, Turkey, p. 255.

[inz212820-bib-0009] Böhme M , Spassov N , Ebner M *et al.* (2017). Messinian age and savannah environment of the possible hominin Graecopithecus from Europe. PLoS ONE 12, e0177347. 28531204 10.1371/journal.pone.0177347 PMC5439672

[inz212820-bib-0011] Colombero S , Pavia M , Carnevale G (2015). Old World porcupine (Rodentia, Hystricidae) remains from The Late Messinian of Piedmont, NW Italy. Rivista Italiana Di Paleontologia E Stratigrafia 121, 243–253.

[inz212820-bib-0012] Coppola F , Cilia G , Bertelloni F *et al*. (2020). Crested porcupine (*Hystrix cristata* L.): A new potential host for pathogenic Leptospira among semi‐fossorial mammals. Comparative Immunology, Microbiology and Infectious Diseases 70, 101472. 32208192 10.1016/j.cimid.2020.101472

[inz212820-bib-0013] Corbet GB , Jones LA (1965). The specific characters of the crested porcupines, subgenus *Hystrix* . Proceedings of the Zoological Society of London 144, 285–300.

[inz212820-bib-0014] Czernielewski M (2023). A new species of *Hystrix* (Rodentia: Hystricidae) from the Pliocene site of Węże 1 in southern Poland. Acta Geologica Polonica 73, 73–83.

[inz212820-bib-0015] De Bonis L , Bouvrain G , Geraads D , Koufos G (1992). A skull of *Hystrix primigenia* from the late Miocene of Macedonia (Greece). Neues Jahrbuch für Geologie und Paläontologie ‐ Monatshefte 2, 75–87.

[inz212820-bib-0081] Ercan T , Dinçel A , Metin S , Turkecan A , Günay A (1978). Geology of the Neogene basins in Uşak region. Bulletin of the Geological Society of Turkey 21, 97–106.

[inz212820-bib-0017] Fejfar O , Sabol M (2022). Pliocene vertebrates from Ivanovce and Hajnácka (Slovakia). XI. Fossil record of porcupines (Hystricidae) from Ivanovce. Neues Jahrbuch für Geologie und Paläontologie ‐ Abhandlungen 305, 97–108.

[inz212820-bib-0018] Festani AB , Pavlakis PP , Symeonides N (1997). First discovery of *Hystrix primigenia* Wagner from the Late Miocene to Early Pliocene deposits of Shahinova, Berat, South‐West Albania. Annalen des Naturhistorischen Museums in Wien 98, 155–172.

[inz212820-bib-0019] Flynn LJ , Wu WY (2017). The bamboo rats and porcupines of Yushe Basin. In: Flynn JL , Wu WY , eds. Late Cenozoic Yushe Basin, Shanxi Province, China: Geology and Fossil Mammals. Vertebrate Paleobiology and Paleoanthropology Series. Springer, Dordrecht. 10.1007/978-94-024-1050-1_15

[inz212820-bib-0084] Garevski R (1956). Neue fundstellen von pikermifauna in Mazedonien. Acta Musei Macedonici Scientiarum Naturalium Skopje 4, 67–96

[inz212820-bib-0085] Gaudry A (1862). *Animaux Fossiles et Géologie de l’Attique*: d’après les recherchers faites en 1855–56 et an 1860. F. Savy Editeur, Paris.

[inz212820-bib-0022] Geraads D , Spassov N , Hristova L , Markov GN , Tzankov T (2011). Upper Miocene mammals from Strumyani, South‐Western Bulgaria. Geodiversitas 33, 451–484.

[inz212820-bib-0023] Halaçlar K , Erol AS , Köroglu T , Rummy P , Deng T , Mayda S (2024). A new Late Miocene *Hystrix* (Hystricidae, Rodentia) from Turkey. Integrative Zoology 19, 548–563. 10.1111/1749-4877.12754 37532680

[inz212820-bib-0079] Hammer O , Harper DAT , Ryan PD (2001). PAST: Paleontological statistics software package for education and data analysis. Palaeontologia Electronica 4, 9.

[inz212820-bib-0025] Happold DCD (2013). Mammals of Africa, volume III: Rodents, Hares and Rabbits. Bloomsbury Publishing, London.

[inz212820-bib-0026] Hürzeler J , Engesser B (1976). Les faunes mammifères néogènes du Bassin de Baccinello (Grosseto, Italie). Comptes Rendus de la Academie des Sciences Paris 283, 333–336.

[inz212820-bib-0027] Karaoğlu Ö , Helvacı C , Ersoy Y (2010). Petrogenesis and ^40^Ar/^39^Ar geochronology of the volcanic rocks of the Uşak‐Güre basin, western Türkiye. Lithos 119, 193–210.

[inz212820-bib-0028] Kormos T (1911). A Polgardi Pliocen csonlelet. Földtani Közlöny 61, 46–64.

[inz212820-bib-0030] Koufos GD , Kostopoulos DS , Vlachou TD , Konidaris GE (2011). A synopsis of the late Miocene Mammal Fauna of Samos Island, Aegean Sea, Greece. Geobios 44, 237–251.

[inz212820-bib-0029] Koufos GD (2023). Updating the Fauna and Age of the Neogene‐Quaternary Large Mammal Sites of Greece. Available from URL: 10.2139/ssrn.4545021

[inz212820-bib-0031] Kovachev D (2012). A porcupine skeleton of *Hystrix* (*Hystrix*) *primigenia* (Wagner) from the Upper Maeotian (Turolian) near Hadzhidimovo, SW Bulgaria. Geologica Balcanica 41, 3–20.

[inz212820-bib-0032] Kretzoi M (1951). The Hipparion Fauna from Csakvar. Foldtani Kozlony 81, 384–417.

[inz212820-bib-0033] Lazaridis G , Tsoukala E , Rae TC , Gómez‐Olivencia A , Nagel D , Bartsiokas A (2018). *Mesopithecus pentelicus* from the Turolian locality of Kryopigi (Kassandra, Chalkidiki, Greece). Journal of Human Evolution 121, 128–146. 29754742 10.1016/j.jhevol.2018.04.003

[inz212820-bib-0034] Lopatin AV , Tesakov AS , Titov VV (2003). Late Miocene early Pliocene porcupines (Rodentia, Hystricidae) from south European Russia. Russian Journal of Theriology 2, 26–32.

[inz212820-bib-0035] Lydekker R (1878). Notices of Siwalik mammals. Records of the Geological Survey of India 11, 64–104.

[inz212820-bib-0036] Massini F , Rook L (1993). *Hystrix primigenia* (Mammalia, Rodentia) from the Late Messinian of the Monticino gypsum quarry (Faenza, Italy). Bolletino della Società Paleontologica Italiana 32, 79–87.

[inz212820-bib-0037] Mein P , Moissenet E , Adrover R (1990). Biostratigraphie du Néogène supérieur de Teruel. Paleontologia i Evolució 23, 121–139.

[inz212820-bib-0039] Melentis J , Schneider H (1966). Eine neue Pikermifauna in der Nahe der Ortschaft Alifaka in Thessalien. Annales Géologiques des Pays Helléniques 17, 267–288.

[inz212820-bib-0038] Melentis JK (1967). Studien über fossile Vertebraten Griechenlands. 19. Die Pikermifauna von Halmyropotamos (Euböa Griechenlands). I Teil: Odontologie und Kraniologie. Annales Géologiques des Pays Helleniques 19, 283–411

[inz212820-bib-0041] Mori E , Ancillotto L , Lovari S *et al.* (2019). Skull shape and Bergmann's rule in mammals: hints from Old World porcupines. Journal of Zoology 308, 47–55.

[inz212820-bib-0040] Mori E , Maggini I , Menchetti M (2014). When quills kill: The defense strategy of the crested porcupine *Hystrix cristata* L., 1758. Mammalia 78, 229–234.

[inz212820-bib-0043] Nishioka Y , Zin‐Maung‐Maung‐Thein EN , Tsubamoto T , Nishimura T , Ito T , Thaung‐Htike TM (2011). New *Hystrix* (Mammalia, Rodentia) from the Late Miocene/Early Pliocene of Myanmar. Journal of Vertebrate Paleontology 31, 919–924.

[inz212820-bib-0044] NOW Community (2023). New and Old Worlds Database of Fossil Mammals (NOW). Licensed under CC BY 4.0. Available from URL: https://nowdatabase.org/now/database/

[inz212820-bib-0046] Ozansoy F (1969). Ege fosil omurgalı faunaları ve Hipparion'lu faunaların dikey yayılımı. Maden Tetkik ve Arama Dergisi 72, 189–193.

[inz212820-bib-0045] Ozansoy F (1957). Faunes de Mammiferes du Tertiaire de Turquie et leurs revisions stratigraphiques. Bulletin of the Mineral Research and Exploration Institute of Turkey 49, 29–48.

[inz212820-bib-0047] Prieto J (2013). *Hystrix* record from Taşkınpaşa (Upper Miocene, Central Anatolia). Zitteliana A 53, 179–180.

[inz212820-bib-0048] Riabinin AN (1929). Faune de mammiferes de Taraklia. I. Carnivora vera, Rodentia, Subungulata. Travaux de Musee Geologique pres l'Academie des Sciences de l'URSS 5, 75–134.

[inz212820-bib-0049] Şen S (1994). Les gisements de mammifères du Miocène supérieur de Kemiklitepe, Turquie: 5. Rongeurs, Tubulidentés et Chalicothères. Bulletin du Muséum National d'Histoire Naturelle. Section C 16, 97–110.

[inz212820-bib-0050] Şen S (1999). Family Hystricidae. In: Rossner E , Heissig K , eds. The Miocene Land Mammals of Europe. Verlag Dr. Friedrich Pfeil, Munchen, pp. 427–434.

[inz212820-bib-0051] Şen S (2001a). Rodents and insectivores from the Upper Miocene of Molayan, Afghanistan. Palaeontology 44, 913–932.

[inz212820-bib-0052] Şen S (2001b). Early Pliocene porcupine (Mammalia, Rodentia) from Perpignan, France: A new systematic study. Geodiversitas 23, 303–312.

[inz212820-bib-0082] Şen S , De Bonis L , Dalfes N , Geraads D , Koufos G (1994). Les gisements de mammifères du Miocène supérieur de Kemiklitepe, Turquie: 1. Stratigraphie et magnétostratigraphie. Bulletin du Muséum National d'Histoire Naturelle Paris 16, 5–17.

[inz212820-bib-0053] Şen S , Kovatchev DB (1987). The porcupine *Hystrix primigenia* (WAGNER) from the Late Miocene of Bulgaria. Proceedings of the Koninklijke Nederlandse Akademie van Wetenschappen B 90, 317–323

[inz212820-bib-0055] Şen S , Purabrishemi Z (2010). First porcupine fossils (Mammalia, Rodentia) from the late Miocene of NW Iran, with notes on late Miocene–Pliocene dispersal of porcupines. Paläontologische Zeitschrift 84, 239–248.

[inz212820-bib-0056] Seyitoğlu G , Alçiçek MC , Işık V *et al.* (2009). The stratigraphical position of Kemiklitepe fossil locality (Eşme, Uşak) revised: Implications for the Late Cenozoic sedimentary basin development and extensional tectonics in western Türkiye. Neues Jahrbuch für Geologie und Paläeontologie 251, 1–15.

[inz212820-bib-0057] Solounias N (1981). Mammalian fossils of Samos and Pikermi. Part 2. Resurrection of a classic Turolian fauna. Annals of the Carnegie Museum 50, 231–270.

[inz212820-bib-0058] Spassov N , Geraads D , Hristova L *et al*. (2012). A hominid tooth from Bulgaria: The last pre‐human hominid of continental Europe. Journal of Human Evolution 62, 138–145. 22153571 10.1016/j.jhevol.2011.10.008

[inz212820-bib-0059] Spassov N , Geraads D , Hristova L , Markov GN , Garevska B , Garevski R (2018). The late Miocene mammal faunas of the Republic of Macedonia (FYROM). Palaeontographica, Abteilung A: Palaozoologie ‐ Stratigraphie 311, 1–85.

[inz212820-bib-0060] Sulimski A (1960). *Hystrix primigenia* (Wagner) in the Pliocene fauna from Weze. Acta Palaeontologica Polonica 5, 319–336.

[inz212820-bib-0061] Tuna V (1985). Kemiklitepe (Usak, Esme) Omurgali faunasi Hipparionlarinda odontolojik degisimler. Türkiye Jeoloji Kurumu Bülteni 28, 47–54.

[inz212820-bib-0063] Ünay E , Bruijn HD (1984). On some Neogene rodent assemblages from both sides of the Dardanelles, Turkey. Newsletters in Stratigraphy 13, 119–132.

[inz212820-bib-0062] Uldis R (2012). Porcupines: The Animal Answer Guide. Johns Hopkins University Press, Baltimore. 10.1353/book.18820

[inz212820-bib-0064] Van Weers DJ (1990). Dimensions and occlusal pattern in molars of *Hystrix brachyura* Linnaeus, 1758 (Mammalia, Rodentia) in a system of wear categories. Bijdragen tot de Dierkunde 60, 121–134.

[inz212820-bib-0065] Van Weers DJ (2004). Comparison of Neogene low crowned *Hystrix* species (Mammalia, Porcupines, Rodentia) from Europe, West and Southeast Asia. Beaufortia 54, 75–80.

[inz212820-bib-0066] Van Weers DJ (2005). A taxonomic revision of the Pleistocene *Hystrix* (Hystricidae, Rodentia) from Eurasia with notes on the evolution of the family. Contributions to Zoology 74, 301–312.

[inz212820-bib-0067] Van Weers DJ , Montoya P (1996). Taxonomy and stratigraphic record of the oldest European porcupine *Hystrix parvae* (Kretzoi, 1951). Proceedings of the Koninklijke Nederlandse Akademie van Wetenschappen 99, 131–141.

[inz212820-bib-0068] Van Weers DJ , Rook L (2003). Turolian and Ruscinian porcupines (genus *Hystrix*, Rodentia) from Europe, Asia and North Africa. Paläontologische Zeitschrift 77, 95–113.

[inz212820-bib-0069] Vekua AK (1972). *The Kwabeb Vertebrate Fauna of Akchag*. Gruzinskaya Akademiya Nauk, Moskva.

[inz212820-bib-0070] Viviano A , Amori G , Luiselli L , Oebel H , Bahleman F , Mori E (2020). Blessing the rains down in Africa: Spatiotemporal behaviour of the crested porcupine *Hystrix cristata* (Mammalia: Rodentia) in the rainy and dry seasons, in the African savannah. Tropical Zoology 33, 113–124.

[inz212820-bib-0071] Wagner A (1848). Urweltliche Säugetier‐Überreste aus Griechenland. Abhandlungen der Mathematisch Physikalischen Classe der Königlich‐Bayerischen Akademie der Wissenschaften 5, 333–510.

[inz212820-bib-0072] Wang BY , Qi GQ (2005). A porcupine (Rodentia, Mammalia) from Lufengpithecus site, Lufeng, Yunnan. Vertebrata PalAsiatica 43, 11–23. (In Chinese.)

[inz212820-bib-0073] Wang BY , Qiu ZX (2002). A porcupine from Late Miocene of Linxia basin, Gansu, China. Vertebrata PalAsiatica 40, 23–33. (In Chinese.)

[inz212820-bib-0074] Wang BY , Qiu ZX (2020). New *Hystrix* (Hystricidae, Rodentia) from the Neogene of Linxia Basin, Gansu, China. Vertebrata PalAsiatica 58, 204–220.

[inz212820-bib-0075] Więckowski W , Cohen S , Mienis HK , Horwitz L (2013). The excavation and analysis of porcupine dens and burrowing on ancient and recent faunal and human remains at Tel Zahara (Israel). Bioarchaeology of the Near East 7, 3–20.

[inz212820-bib-0076] Xafis A , Mayda S , Alçiçek MC *et al.* (2021). Large giraffids (Mammalia, Ruminantia) from the new late Miocene fossiliferous locality of Kemiklitepe‐E (Western Anatolia, Turkey). Palaeobiodiversity and Palaeoenvironments 101, 853–867. 34721707 10.1007/s12549-020-00433-4 PMC8550776

[inz212820-bib-0077] Yalçınlar I (1946). Une faune de vertébrés miocènes aux environs d'Esme (Turquie, vallée du Méandre supérieur). Istanbul Üniversitesi Fen Fakültesi Mecmuasi 11, 124–130.

