# Supplementary figures and images for: A newly discovered Hystrix primigenia specimen from the Kemiklitepe collection at Ege University Natural History Museum: insights into paleobiogeography in Eurasia
Source: Integr Zool. 2024 Apr 3;20(1):73–87. doi: 10.1111/1749-4877.12820 (PMC11693979; doi:10.1111/1749-4877.12820)

## Slide 1
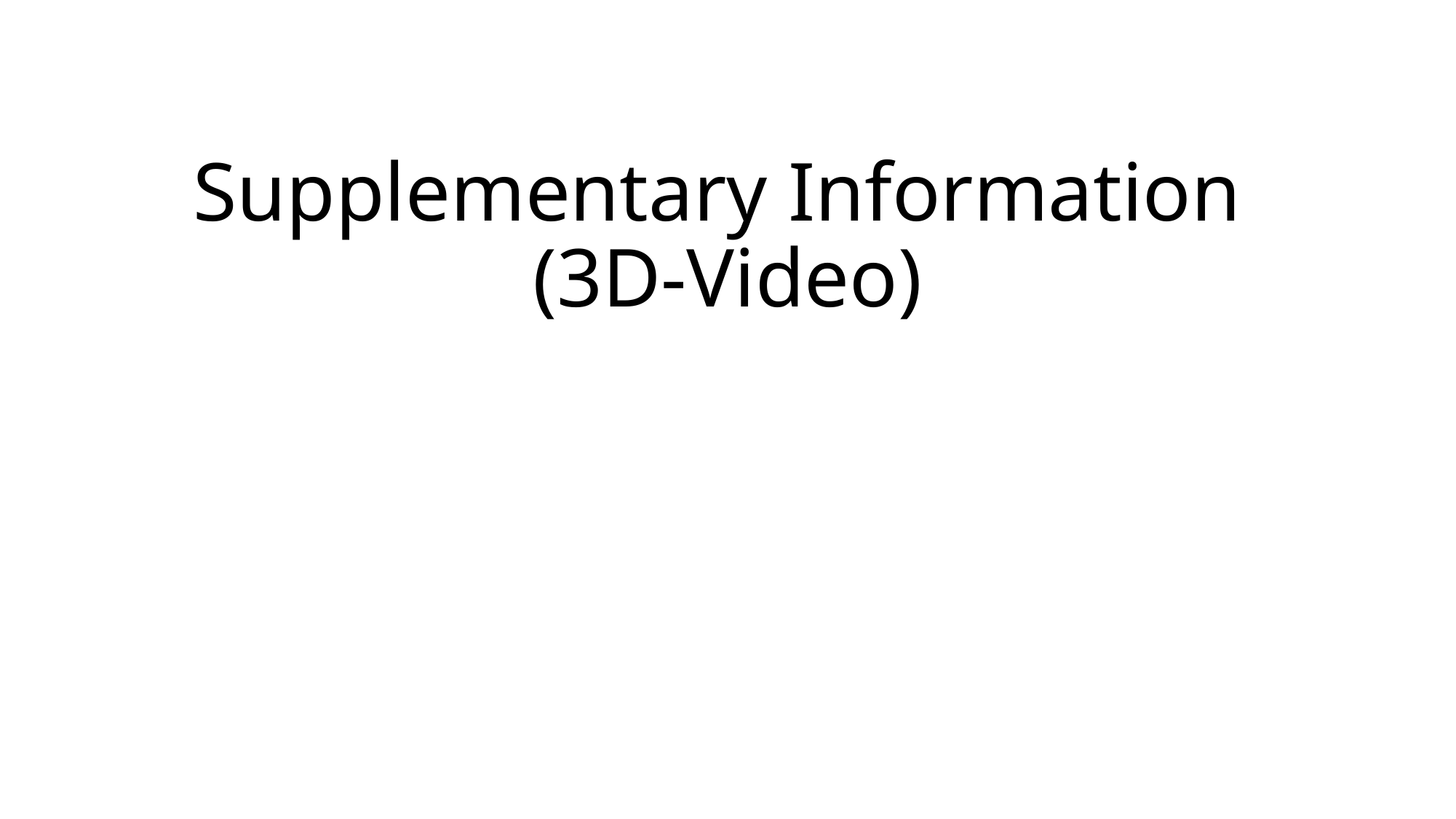

# Supplementary Information (3D-Video)

## Slide 2
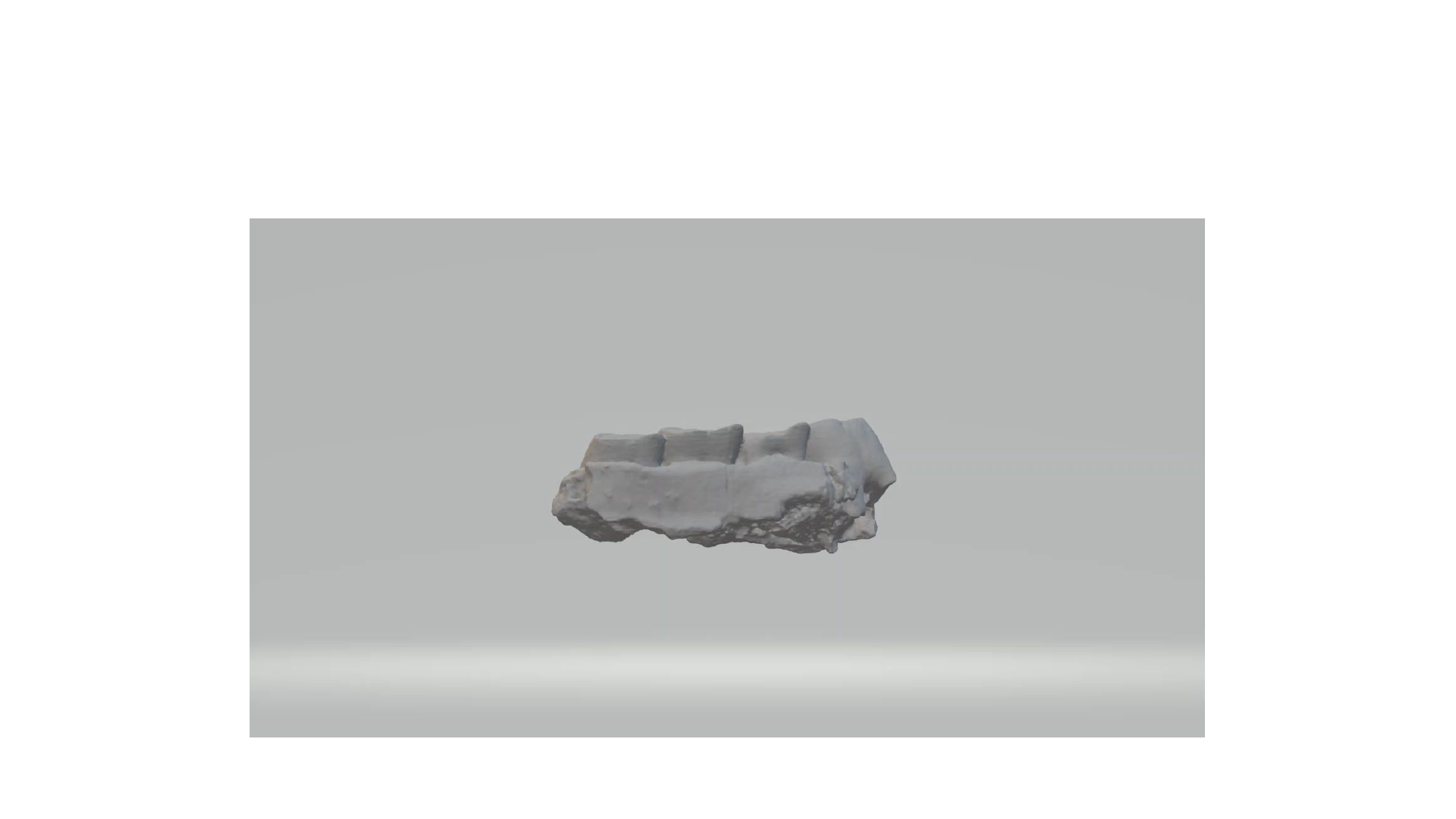

#

## Slide 3
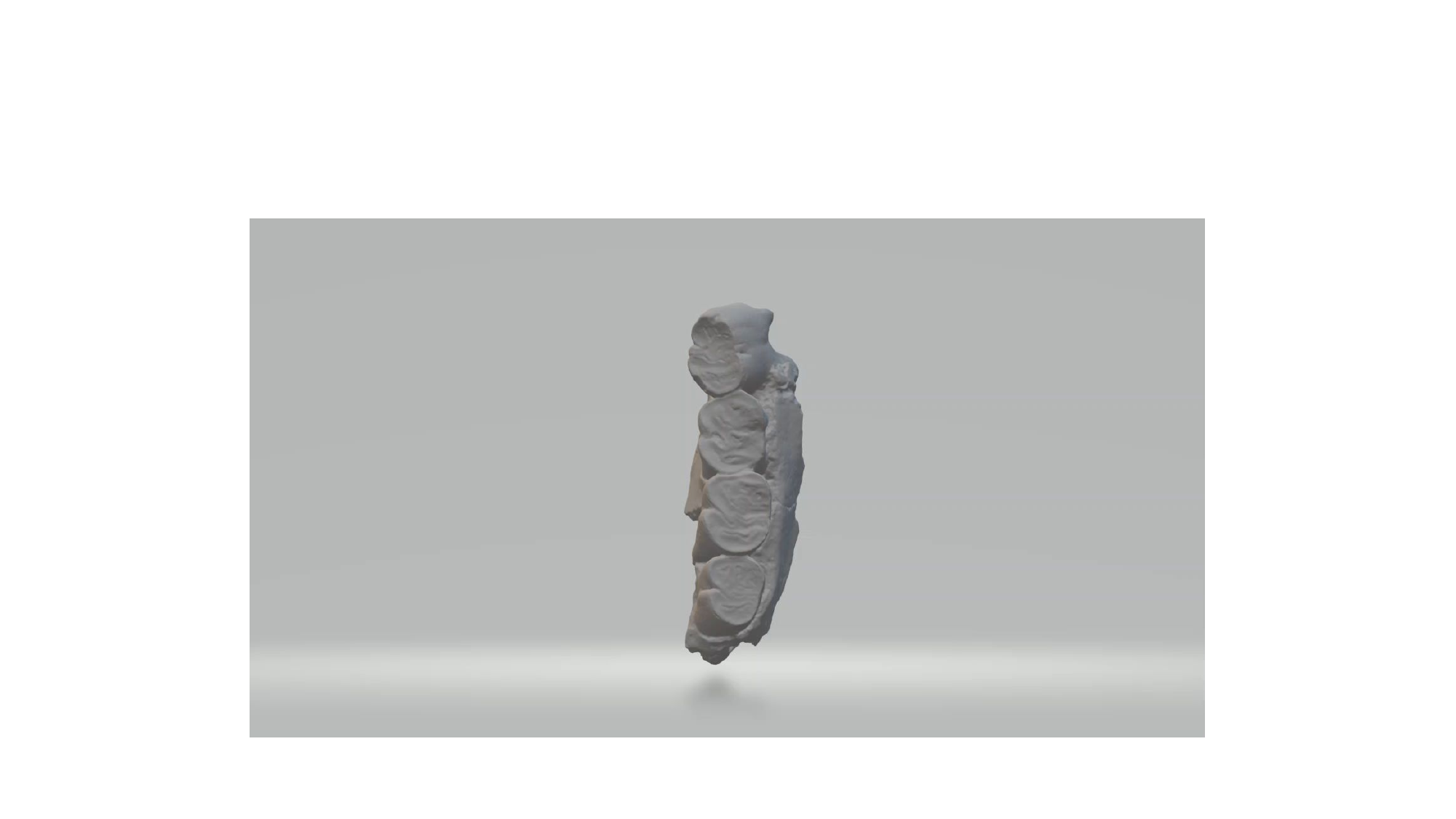

#

Supplement: Supplementary file 1 — Supplementary Materials [file INZ2-20-73-s001.pptx]
